# COVID’s collateral damage: likelihood of measles resurgence in the United States

**DOI:** 10.1186/s12879-022-07703-w

**Published:** 2022-09-20

**Authors:** Mugdha Thakur, Richard Zhou, Mukundan Mohan, Achla Marathe, Jiangzhuo Chen, Stefan Hoops, Dustin Machi, Bryan Lewis, Anil Vullikanti

**Affiliations:** Biocomplexity Institute, Town Center Four, 994 Research Park Boulevard, Charlottesville, VA 22904 USA

**Keywords:** MMR vaccination, Home isolation, Social network, Network epidemiology, Vulnerable populations, Health equity, Agent-based model, NIS

## Abstract

**Background:**

Lockdowns imposed throughout the US to control the COVID-19 pandemic led to a decline in all routine immunizations rates, including the MMR (measles, mumps, rubella) vaccine. It is feared that post-lockdown, these reduced MMR rates will lead to a resurgence of measles.

**Methods:**

To measure the potential impact of reduced MMR vaccination rates on measles outbreak, this research examines several counterfactual scenarios in pre-COVID-19 and post-COVID-19 era. An agent-based modeling framework is used to simulate the spread of measles on a synthetic yet realistic social network of Virginia. The change in vulnerability of various communities to measles due to reduced MMR rate is analyzed.

**Results:**

Results show that a decrease in vaccination rate $(\mathrm{\alpha })$ has a highly non-linear effect on the number of measles cases and this effect grows exponentially beyond a threshold $(\mathrm{\alpha })$. At low vaccination rates, faster isolation of cases and higher compliance to home-isolation are not enough to control the outbreak. The overall impact on urban and rural counties is proportional to their population size but the younger children, African Americans and American Indians are disproportionately infected and hence are more vulnerable to the reduction in the vaccination rate.

**Conclusions:**

At low vaccination rates, broader interventions are needed to control the outbreak. Identifying the cause of the decline in vaccination rates (e.g., low income) can help design targeted interventions which can dampen the disproportional impact on more vulnerable populations and reduce disparities in health. Per capita burden of the potential measles resurgence is equivalent in the rural and the urban communities and hence proportionally equitable public health resources should be allocated to rural regions.

## Background

Severe symptoms and high transmissibility are well known characteristics of measles. Although measles is preventable by the measles, mumps, and rubella (MMR) vaccine, high level of immunization rate (more than 95%) is required to prevent outbreaks through herd immunity. Measles was declared eliminated from the US in 2000; however, due to increasing vaccine hesitancy, various states have seen measles outbreaks in the last two decades [1].

The 2019 measles outbreak in the US (the largest one reported since 1994) constituted of imported cases, majority of which were unvaccinated US resident global travelers [2]; 85% of the cases occurred in close-knit under-immunized communities [2]. In the light of increased mobility in this century, any under-immunized region is at a risk of a measles outbreak due to case importation.

During the COVID-19 pandemic, routine immunizations have reportedly decreased in various parts of the world, including in the USA [3–7]. Globally, over 27 million children are estimated to have missed the first dose of the measles vaccine, just in 2020 [7]. With the decline in the routine immunizations, the end of social-distancing and lockdown will likely result in a surge in highly contagious diseases like measles [8–11]. Our goal is to assess the effect of reduction in the MMR vaccination rates on the potential outbreak of measles in the post-lockdown COVID-19 era. Prior studies, such as [3–7], only focus on the reduction in coverage rates; disease transmission models are needed for estimating the risk of outbreaks. Gaythorpe et al. [10] use a modeling approach to estimate the health impacts of 50% reduction in vaccination coverage in 2020 and delays in vaccination campaign in 10 countries, and report that there could be significant risk of measles in some countries. However, their models consider limited details of population mixing, which makes it difficult to understand the impact of individual level contacts and behaviors, especially of children in schools.

Along with individuals’ compliance to vaccination and distancing decisions, the course of an outbreak is impacted by the location of their residence. We use our model to analyze the differences in the likelihood and the burden of a Measles outbreak in the urban and the rural regions and discuss the implications.

In this research, we examine the risk of measles in a highly resolved agent-based model, which incorporates detailed contacts, including at schools. We study the impact of different levels of immunization reduction and seeding of the outbreak in rural versus urban regions, and observe the effect of location on outcome metrics. We simulate a variety of counterfactual scenarios which consider different seeding locations, disease transmission levels, compliance to stay-home intervention, spatial distribution of immunization rates, and various levels of reduction in MMR immunization rate. The results (1) show the effect of reduced immunization rates on measles’ incidence, (2) compare the outbreaks when the imported case is in a rural region versus urban region for various levels of reduction in the vaccination rate, (3) analyze the proportional impact on the rural and urban communities, (4) identify the communities that are disproportionately affected, and (5) assess the effect of interventions.

## Methods

**Fig. 1:**
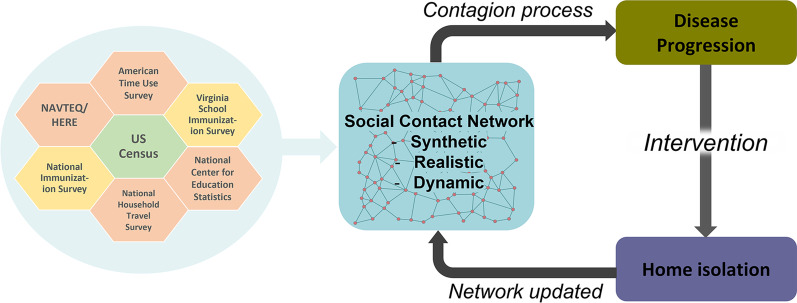
Design and flow of the model. Summary of the design and the flow of the model. Green hexagon (source for synthetic population), orange hexagons (sources for activity-based contact network) and yellow hexagons (sources for MMR immunization rate) show select sources of input data

We simulate the transmission of measles in Virginia in two scenarios—(1) pre-COVID-19 (base-case scenario), and (2) post-lockdowns (i.e. after resuming social activities to pre-covid levels but reduced MMR immunization rate) to quantify the increased risk of measles resurgence post-COVID due to the decline in routine immunizations. The transmission process is characterized by an agent-based modeling framework implemented on a synthetic yet realistic activity-based social network (discussed below), which has been used in several modeling studies, such as [12–14]. The MMR vaccination status of the children in the synthetic population is determined from the school immunization data publicly available from the Virginia Department of Health. The vaccination status of adults is determined by the state-level immunization rate available from the US Centers for Disease Control and Prevention (CDC). Figure 1 graphically shows the different components of the model and the methods and the flow of processes between them. In the following subsections we describe the process used for constructing the synthetic network, the datasets, and the procedure for assigning the immunization status to individuals, the disease transmission model, the interventions, and the experimental parameters.

### Synthetic population and contact network

The social contact network of the Virginia population consists of more than 7.6 million nodes and 371.9 million edges, and was developed using a “first principles" approach [12, 13, 15, 16]. The nodes represent synthetic individuals with respective households which are located geographically. Each node is endowed with features like age, race, gender, household size, household location, household income etc., as available in the US Census.

The synthetic population is created by integrating various datasets from commercial and public sources into a common architecture for data exchange. Each synthetic individual is placed in a household with other synthetic people, and each household is located geographically in such a way that a census of the synthetic population yields results statistically indistinguishable from the original census data, if they are both aggregated to the block group level [17, 18]. Further, counties of Virginia are designated as “urban” or “rural” based on the US Census Urban and Rural Classification [19].

Synthetic individuals are assigned daily activities using time-use surveys (American Time Use Survey data [20], National Household Travel Survey Data [21] and Multinational Time Use Study [22]), and then assigned a geo-location for each activity that each person performs. The geo-locations are based on data from HERE/NAVTEQ,^1^National Center for Education Statistics,^2^LandScan,^3^OpenStreetMap^4^ etc. Finally, a dynamic social bipartite visitation network is constructed when people visit locations to perform daily activities and come in physical contact with others at those locations. The colocation based social contact network is used for the spread of disease transmission. For more details on the social network construction, see [12, 15, 17, 23].

### Disease transmission model

**Fig. 2:**
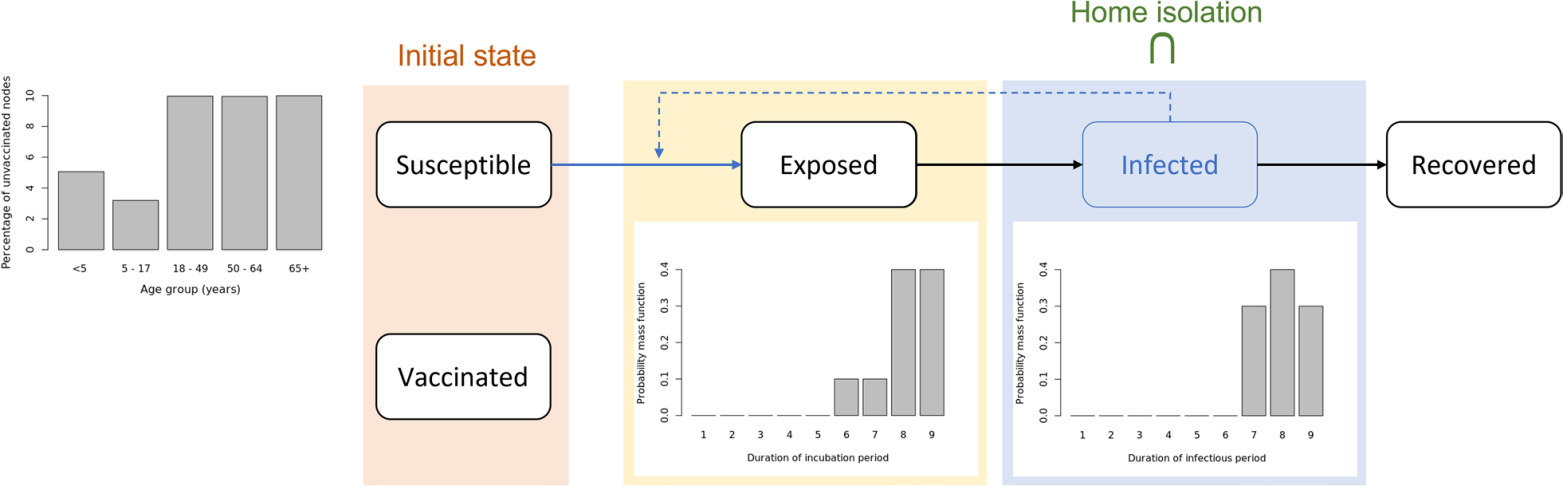
Disease transmission flowchart. SEIR disease transmission model for measles. Only susceptible nodes can become exposed and infected. Exposed is the latent stage of Measles and Infected state comprises of the infectious period (presymptomatic incubation and rash)

We use an agent-based disease transmission model to simulate the spread of measles in the population. The model computes probabilistic disease transmission between individuals (nodes in the network) as well as keeps track of the disease progression and the different health states of each node, as shown in Fig. 2. The evolution of the health state of any individual is assumed to follow a network-based SEIR (Susceptible-Exposed-Infected-Recovered) model.

The simulation is initialized by (a) setting the health states of all the immunized nodes as “Vaccinated", (b) selecting a random node from age group 5–17 years as “Infected" (and infectious) to seed the epidemic, and (c) setting all other nodes as “Susceptible". We assume that the vaccine is perfect, and thus no vaccinated node contracts the disease [24]. The propensity of transmission, i.e., a susceptible node contracting the disease while in contact with (that is, sharing an edge with) an infectious node, is calculated by summing for all edges of the susceptible node, the product of the contact duration over a day (24 h), susceptibility ($\upsigma$) of the susceptible node, infectivity ($\upiota$) of a contact node and transmissibility ($\uptau$) for each edge.

Thus, if the set of health states is $\upchi =\{\mathrm{Susceptible},$ $\mathrm{Exposed},$ $\mathrm{Infected},$ $\mathrm{Recovered}\}$, the associated susceptibility and infectivity for a node $\mathrm{P}$ in the population is denoted by ${\upsigma }_{\mathrm{P}}(\mathrm{X})$ and ${\upiota }_{\mathrm{P}}(\mathrm{X})$ respectively for $\mathrm{X}\in\upchi$. We assume that susceptibility of “Susceptible" health state is 1, and 0 for all other health states and infectivity of “Infected" health state is 1, and 0 for all other health states ensuring that only infected nodes can transmit the disease to a susceptible node. The probability that a susceptible node P becomes exposed by contact with infectious neighbors P’ is a function of the transmissibility $\uptau$ and the contact duration. Notice that the Exposed state stands for the latent period and hence not infectious; the Infected state comprises of the pre-symptomatic and the rash period, and is infectious.

Between September 2018 to August 2019, the measles outbreak in New York city (NYC) led to a total of 649 case counts [2]. Being the latest large outbreak with a population size close to Virginia’s, we chose the transmissibility ($\uptau$) value to be 0.5 which on an average, with one node initially infected between the ages 5–17 years, results in an outbreak of the size proportional to the one that occurred in NYC. Details of the calibration of the parameter $\uptau$ are described in the Appendix.

Once a node becomes exposed, the disease progresses as follows. The maximum duration of the latency period is 9 days and follows the discrete probability distribution {0, 0, 0, 0, 0, 0.1, 0.2, 0.6, 1} of the health state of the node changing to infected. The infectious duration (includes the presymptomatic incubation and the rash periods) of each infected node is then determined by the discrete probability distribution {0, 0, 0, 0, 0, 0, 0.3, 0.7, 1} of the node getting recovered [25] as shown in Fig. 2. Note that, the cumulative probability distribution is reported as {${\mathrm{p}}_{1},{\mathrm{p}}_{2}\dots ,{\mathrm{p}}_{\mathrm{n}}$}, where ${\mathrm{p}}_{\mathrm{i}}$ is the probability that $\mathrm{i}$ is the last day of latency and of infectiousness respectively; and a measles case will change state within $\mathrm{n}$ days. We assume that a recovered individual obtains permanent immunity against measles. This assumption is realistic for the scope of this paper since we simulate the transmission for a relatively short time duration (365 days). The disease progression related parameters used in the model are described in the Table 1.

**Table 1:** Table of variables. Summary of the variables used in the experiments. ^†^The cumulative probability distribution is reported as {${\mathrm{p}}_{1},{\mathrm{p}}_{2}\dots ,{\mathrm{p}}_{\mathrm{n}}$}, where ${\mathrm{p}}_{\mathrm{i}}$ is the probability that $\mathrm{i}$ is the last day of latency or of infectiousness, and a measles case will change state within $\mathrm{n}$ days

Base case scenario		
Variables	Base case value	Source
Population of Virginia	7,688,059	Synthetic populations [13]
Infected individuals at day 0	1 (5–17 years of age)	Assumed
Proportion vaccinated	91.5%	Calibrated using VDH school immunization data [26] and state-level immunization rate [27]
Transmissibility	0.5	Calibrated to generate an outbreak size of 650 [28]
Simulation duration	365 days	Assumed
Home isolation compliance	90%	[25]
CDF of the latency period distribution	{0, 0, 0, 0, 0, 0.1, 0.2, 0.6, 1}^†^	[25]
CDF of the infectious period distribution	{0, 0,0, 0, 0, 0, 0.3, 0.7,1}^†^	[25]

Experiments	
Variables	Set of values used in experiments
Transmissibility ($\uptau$)	{0.5, 0.6, 0.7, 0.8, 0.9}
Home isolation compliance	{75, 80, 85, 90, 95}%
Home isolation initiation day	{3, 4, 5, 6, 7}
Decline in immunization rate ($\mathrm{\alpha }$)	{0, 5, 10, 15, 20, 25}%

In this study, we assume that over the year of simulation there are no new infant nodes due to birth or nodes removed due to mortality.

### MMR immunization rates

#### Children

Immunization rate for children (up to age 17) was calculated using middle school (6th grade) students’ and kindergarten students’ immunization report for Fall 2018 from the Virginia School Immunization Survey (SIS) which is publicly available at the Virginia Department of Health (VDH) website (https://www.vdh.virginia.gov/immunization/sisresultsarchived/) for public schools. All schools for kindergarten level and schools with less than 10 students for 6th grade level reported only the overall vaccination rate instead of MMR specific immunization rate [26]. We used MMR vaccination rate in the data whenever available and the overall rate otherwise.

The synthetic population network [13] has a record of all the schools included in the National Center for Education Statistics database (Common Core Data^5^ for public schools). We assigned the SIS immunization rate for all the schools common to both SIS data and the synthetic population. Table 2 summarizes the assumptions used for assigning immunization rates to the schools in the synthetic population that were missing in the SIS data. We use latitude and longitude to find the nearest school by Haversine distance measure. We assume that if a household has more than one child of age 14 or below, they all have the same immunization status. The assignment of immunization status is done using a binomial distribution with probability equal to the associated school’s immunization rate (assumptions for different age groups shown in Table 2). The resulting childhood (ages under 18 years) immunization rate for the Virginia population network is 96.331% whereas the overall vaccination rate in the Virginia population is 91.496%. Figures in the Appendix show a county-wise spatial distribution of the immunization rates for the synthetic population in Virginia.

**Table 2:** Immunization rate assumptions. Assumptions for assigning immunization rates to schools and individuals

School	
For schools in synthetic population	Assumption of immunization rate
Both 6th grade and kindergarten rates available	Use the corresponding immunization rates
Kindergarten rate not available	Use the kindergarten rate of the nearest school
6th grade rate not available	Use the 6th grade rate of the nearest school
Neither available	Use the available corresponding rate of the nearest school

Individuals	
Age	Immunization rate assumption
Up to 11 years	Use the associated school’s kindergarten immunization rate
12 – 17 years	Use the associated school’s 6th grade immunization rate
18 years and above	Use the average state level immunization rate for the years 1995 to 2004

#### Adults

The National Immunization Survey reports the state level immunization rate of 19–35-month-olds for the years 1995 to 2017. Since the subjects in the reports from 1995–2004 are adults in the current year (2021), we use a weighted average of the state level aggregated rates from these years [19, 29–38] (from the trend reports which are publicly available at the CDC’s ChildVaxView webpage^6^) to obtain the state level immunization rate to be 90.03%. We assign these immunization rates to the adults in the population (18 + years age) through uniformly sampling per census block group. The resultant overall vaccination rate of the synthetic population turns out to be 91.5% (i.e. adults and children) which is used in the base-case scenario.

### Interventions

Due to high transmissibility, measles patients are recommended to isolate to prevent secondary infections. Therefore, in our simulations, the intervention applied to infectious individuals is home isolation. We assume that, in the base case, 90% of the individuals entering “infectious" class are compliant to home isolation directive [25].

Since the measles’ rash starts around 3–5 days after the non-specific symptoms of fever and cough, we assume that infectious individuals begin home-isolation three days after entering the infectious state (to account for delay in getting a diagnosis and severe symptoms) and continue home isolation for the rest of their infection duration.

Home isolation is implemented in our simulations in the following manner. At the end of every time step (a day), out of the new “infected" nodes, 90% (referred to as home isolation compliance rate) are randomly selected to initiate home isolation on the third day since becoming “infected" by deactivating all their non-home edges in the network. That is, isolating individuals will not contact any individuals other than their household members. Non-home edges are reactivated after the isolating node has recovered to indicate that the activities of that individual have resumed.

### Simulations

We use a tool called EpiHiper [16] to simulate the spread of measles under various scenarios over the social network of Virginia. It is implemented in C +  + /MPI and is scalable to millions of agents via its parallel algorithm, which enables scaling on distributed memory systems [16]. EpiHiper is a network-based epidemiological model designed to study the impact of individual behavior and public health policies on the spread of infectious diseases. Similar methods have been successfully used to study COVID-19 and Influenza and are discussed in detail in [14, 39–41].

Each simulation begins with one random infected node of age 5–17 years and is run for 365 time-steps (365 days). For the base case scenario, we assume that the transmissibility is 0.5, the home isolation compliance rate is 90% [25] and the home-isolation is initiated three days after entering infectious state. We study two types of outcomes: (a) the expected number and distribution of cases (since there is significant variance in the number of cases), and (b) the probability of having a large outbreak. The latter is important, since the expected number of cases is a small fraction due to the relatively high immunization rates in the population, and the high variance in simulation outcomes. We note that computing the probability of large outbreaks is a rare event, and requires a very large number of simulations to estimate well, which is a novel aspect of our study. Table 1 lists all the variables and their corresponding values used in the experiments. The hardware and software requirements for conducting all the simulations are listed in the Appendix.

#### Effect of lockdown

Rate of routine immunizations is believed to have dropped by as much as 40% in some states in the US during COVID-19 era [5]. Now with the end of the lockdown, an increase in social activities could result in a higher spread of infectious diseases if immunization rates continue to stay low. To assess the effect of a reduced MMR vaccination rate during the pandemic [42] on measles resurgence, we model the decline in the MMR rate in the population that is 12 years or younger, by a parameter $\mathrm{\alpha }$ which is chosen to be in the range of 5% to 25%. These nodes are selected (a) uniformly randomly throughout the network, referred to as the uniform scenario, and (b) (inversely) weighted by their per capita household income (to capture the impact on low-income and vulnerable population), referred to as the weighted scenario. Note that the former scenario assumes the decline in vaccination to be proportional to the population density whereas the latter assumes the decline to be negatively correlated to household income to capture the lack of access to health care services for the lower income households.

The resulting immunization rate distributions for the post-COVID scenarios, i.e. for α > 0 both uniform and weighted, are shown by county in figures in the Appendix.

#### Effect of the rural–urban divide

In Virginia, there are approximately thrice as many individuals residing in the urban counties as in rural counties, and urban residents have, on average, about 1.5 times the per capita household income of the rural residents. We study the distributions of the outbreak size when the source of the outbreak is in an urban county versus in a rural county. We analyze the differences in the distributions for various values of $\mathrm{\alpha }$ (decrease in immunization rate) in the two types of regions.

#### Effect of interventions

The goal of public health interventions is to prevent the spread of the disease. Since the exact values for home-isolation compliance rate, initiation of home-isolation and transmissibility are unknown, we analyze the sensitivity of our outcomes to these parameters. Specifically, we analyze the risk of measles outbreak if.

1. the transmissibility varies between 0.5–0.9.
2. the rate of compliance to home isolation varies between 75–95%.
3. the delay in initiation of home isolation varies between 3–7 days.

#### Vulnerable population groups

We define vulnerability as the risk of getting infected in the simulations. To identify groups of people who may be more vulnerable to measles, and disproportionately so than the rest of the population, we chart the distributions of the proportion of infected population by race, age group, ethnicity, household size and household income. Since none of the distributions are normal, we choose to perform a non-parametric Wilcoxon’s Signed Rank Test to see which groups of people (variables) are significantly more or less vulnerable to measles in three scenarios: the base case and the cases of 25% (uniform and weighted) decline in childhood MMR immunization rate.

To quantify the magnitude of the effect of the variables on vulnerability, through the Wilcoxon test, we calculate the effect size corresponding to each variable tested by dividing the absolute (positive) standardized test statistic z by the square root of the sample size. Based on Cohen’s classification for effect sizes [43], we assume a value around 0.2 denotes small effect, around 0.5 a medium effect and around 0.8 a large effect [44].

## Results

**Fig. 3:**
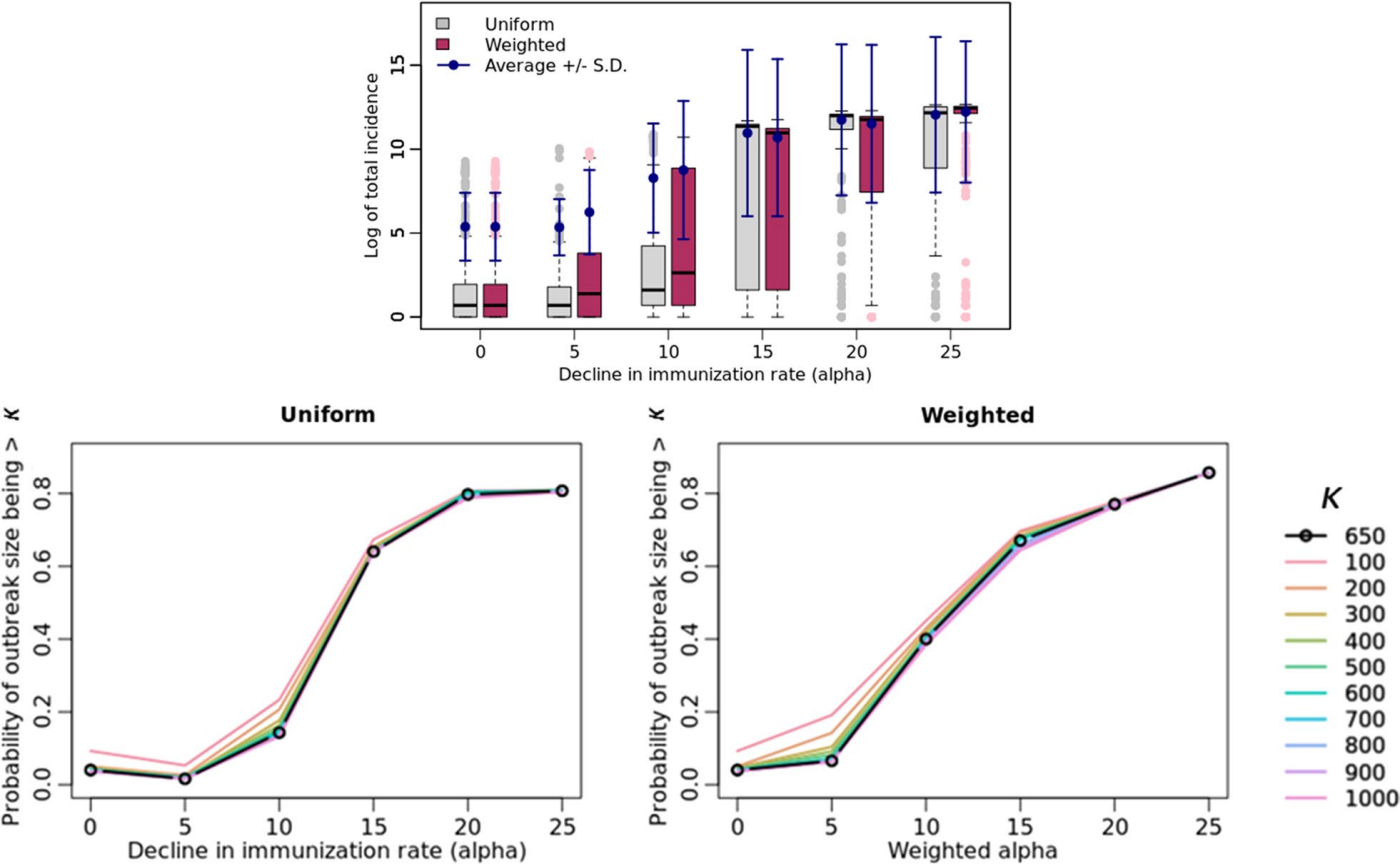
Post-lockdown measles threat. Post-lockdown measles threat as a function of decline in childhood MMR immunization: effect of varying α on the distribution of outbreak on a logarithmic scale, (bottom) the probability of outbreak size bigger than a value $\upkappa$ (in the range of 100 to 1000 where $\upkappa$= 650 represents the probability of outbreak bigger than that of NYC’s). α = 0 indicates the base-case scenario

**Fig. 4:**
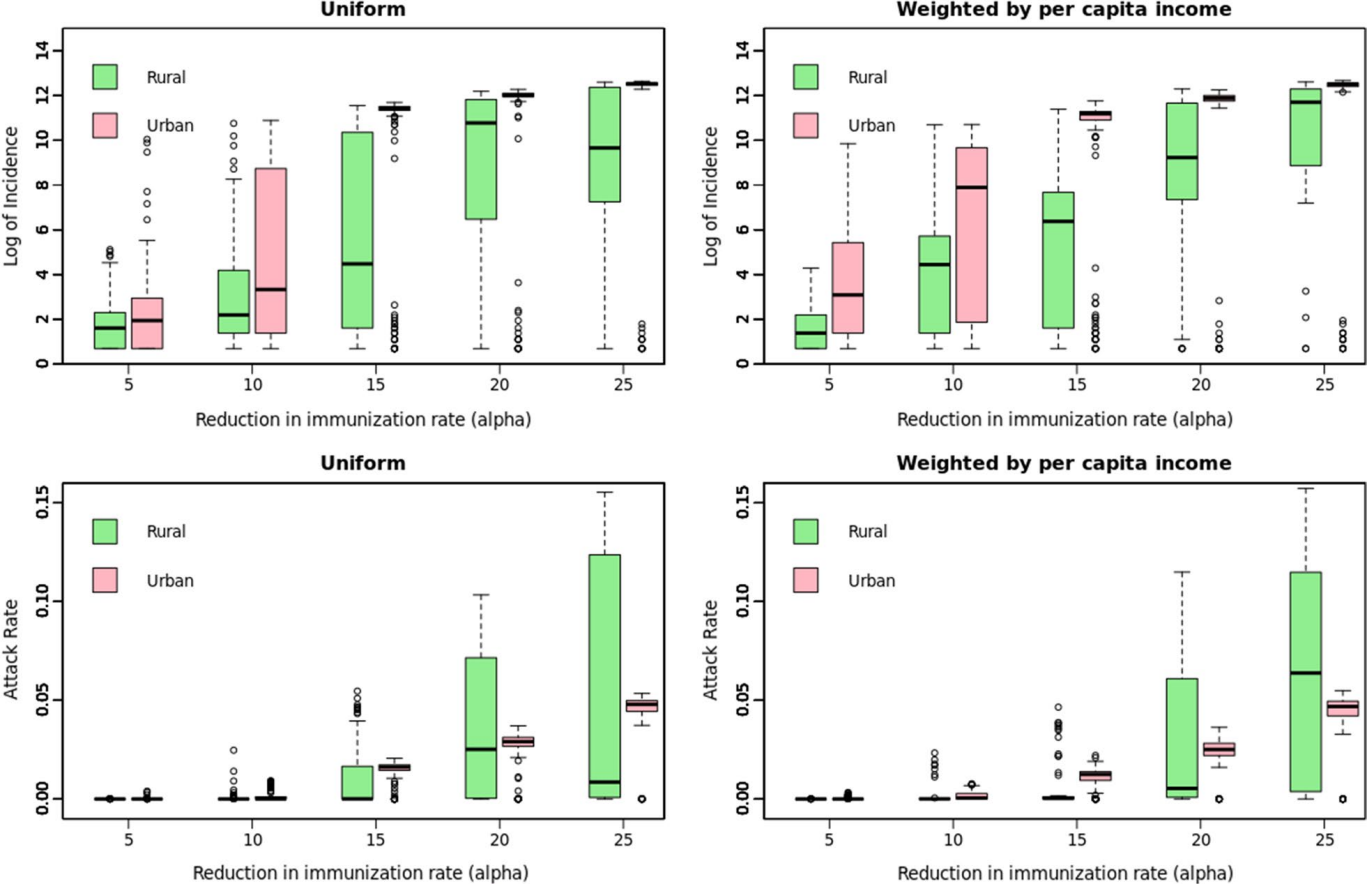
Urban–rural divide. (Left) Uniform, (Right) Weighted, (Top) Logarithm of case counts, (Bottom) Proportion of case counts in rural (out of 1,901,192 individuals) and urban (5,786,867 individuals) regions, respectively

**Fig. 5:**
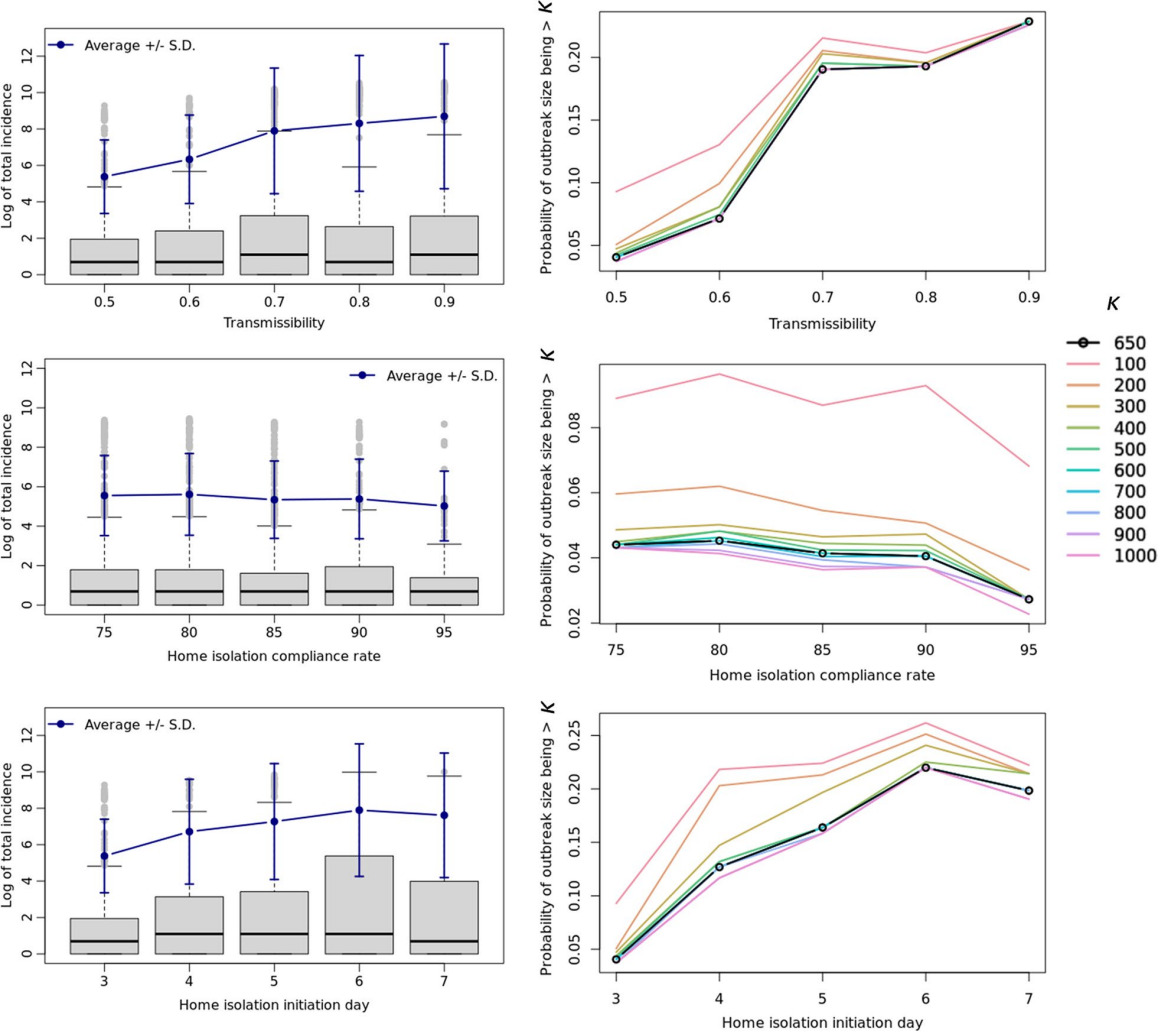
Effect of varying parameters. Effect of varying transmissibility, home isolation compliance rate and the day of initiation of home isolation since becoming infectious in the base case scenario on (left, logarithmic scale) the average measles outbreak size in Virginia, and (right) the probability that the measles outbreak will be bigger than $\upkappa$ (with $\upkappa$ in the range of 100 to 1000). The line for $\upkappa$= 650 represents the probability of outbreak bigger than that of NYC’s

**Fig. 6:**
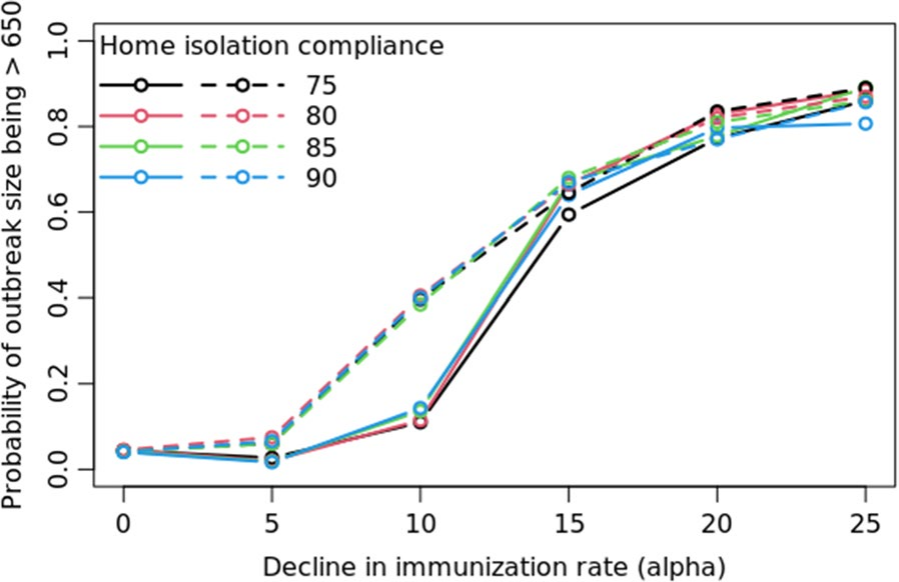
Effect of intervention in post-COVID scenarios. Probability that the measles outbreak will be bigger than NYC’s for varying α (reduction in childhood MMR rate; solid lines are for uniform and dashed for weighted α) for different rates of home isolation compliance. α = 0 indicates the base-case scenario

Figure 3 presents the outcomes for the varying immunization rate scenarios in comparison to the base-case scenario ($\mathrm{\alpha }=0$). Figure 4 shows the distribution of infected cases between rural and urban counties, for the low immunization settings ($\mathrm{\alpha }\in \{5, 10, 15, 20, 25\}$), for fixed values for the parameters of transmissibility (0.5), home isolation compliance rate (90%) and the day of initiating home isolation (3) in the base case. We present a detailed sensitivity analysis of our simulation model in Fig. 5 by varying the parameters one at a time in the base-case scenario. Figure 6 represents the trade-off between the vaccine levels and the compliance rate of home isolation intervention.

### Post-lockdown measles resurgence

While in the base case scenario ($\mathrm{\alpha }=0$), the simulations produce an outbreak of size of a couple of hundred case counts, Fig. 3 shows the impact on the threat of measles outbreak due to the reduced immunization rates in the following two scenarios:

1. Decrease in vaccination rate ($\mathrm{\alpha }$) is uniformly distributed across Virginia (the uniform scenario).
2. Decrease in vaccination rate ($\mathrm{\alpha }$) is correlated to the child’s household’ per capita income (the weighted scenario). This assumes that access to health clinics is inversely proportional to the child’s economic conditions.

The outbreak size grows as the immunization rate decreases in both cases, seemingly exponentially, after a threshold value for $\mathrm{\alpha }$. Notice that for $\mathrm{\alpha }\ge 15\mathrm{\%}$, the average outbreak size increases by many orders of magnitude in both the uniform and weighted scenarios. Figure 3 also shows the probability of having an outbreak larger than the size of the NYC outbreak, i.e., more than 650 infections, under various levels of $\mathrm{\alpha }$.

### Urban–rural divide

As seen from Fig. 4, in high vaccination settings (i.e., lower $\mathrm{\alpha }$), an infection initiated in either a rural or an urban county will lead to equivalent outbreak sizes. In low vaccination settings (i.e., higher $\mathrm{\alpha }$), note that the case counts in urban population is significantly higher than the rural counterparts (top row in Fig. 4). The proportion of the respective population getting infected shows an insignificant difference (bottom row in Fig. 4), but the variance is much larger in the rural regions. Note that the median outbreak size in urban regions increases steeply when $\mathrm{\alpha }$ is between 10 and 15% and the decline in the immunization rate is uniformly distributed.

### Effect of intervention and uncertainty in variables

Figure 5 shows (1) the average change in the outbreak size when transmissibility, compliance to home-isolation and the day home-isolation is initiated, are varied, and (2) the probability of the measles outbreak being bigger than $\upkappa$ case counts, where $\upkappa$ is in the range of 100 to 1000 and $\upkappa \ge 650$ represents the case when the size of the outbreak is bigger than the one that occurred in NYC.

On average, the total incidence does not demonstrate significant sensitivity to compliance to home-isolation, its initiation day, or the transmissibility. In fact, compliance to home-isolation has the least effect on the incidence. Based on Fig. 5, the following conclusions can be drawn:

#### Transmissibility

As the value of $\uptau$ increases beyond 0.5 there is an approximately linear increase in the risk of an outbreak of size bigger than 650, although notice that the increase in the risk is the steepest for $\uptau$ between 0.6 and 0.7.

#### Home isolation compliance

Increasing home isolation compliance up to 95% does not decrease the chance of an outbreak having more than 650 case counts by much, only about 2%.

#### Home isolation initiation day

Delaying the home isolation initiation by an additional day increases the chance of an outbreak that is bigger than 650 case counts, but it peaks by day 6.

Plots for each of the parameters in Fig. 5 show that the probability of large outbreaks start to converge for outbreak size of 400 or more (i.e. $\upkappa \ge 400$.)

#### Trade-off between vaccination rates and the effect of home isolation

Figure 6 shows the effect of varying the home-isolation compliance rates in different immunization settings on the probability of an outbreak size bigger than 650 (size of the NYC outbreak). The magnitude of the effect of the home isolation is very small compared to the impact of decreasing vaccination rates in the population.

#### Vulnerable population groups

**Fig. 7:**
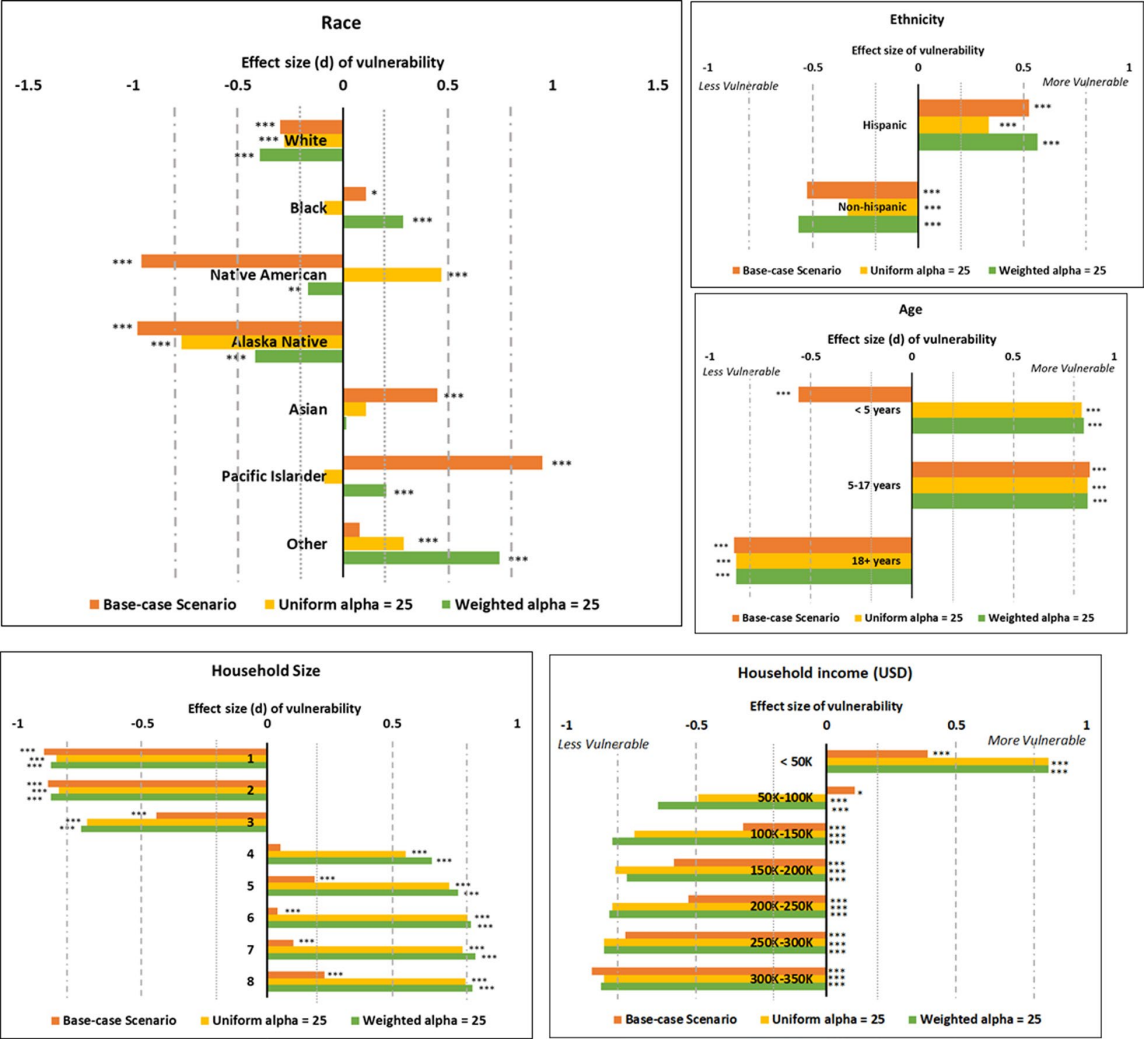
Vulnerability to measles by demographics. The figures show the results of the calculated effect size (d) through the Wilcoxon test. Values of d close to zero imply that the variable’s likelihood of measles burden is the least disproportionate in the population; values around 0.2 denotes small effect, around 0.5 a medium effect and around 0.8 a large effect [43, 44]. *: p < 0.05, **: p < 0.01, ***: p < 0.001

Figure 7 displays the results of Wilcoxon’s Signed Rank Test which compares the vulnerability of the population to measles by race and by age groups with statistical significance level assumed at 0.05. It shows that the White population is always significantly less vulnerable to measles i.e. they are proportionally less represented in the case counts. However, for the African American, Pacific Islander (includes Native Hawaiian) and the Native American population, the incidence level depends on the immunization level in the population as well as the distribution of the reduction in the immunization rate. In low immunization settings, children under the age of 5 years are found to be significantly more vulnerable to infection as compared to the base case. Hispanic population is more vulnerable than the non-Hispanic population in both base-case and low-immunization rate scenarios; however, the effect size is lower in the uniform case than the weighted case and the base-case scenarios.

Households with sizes greater than 3 are consistently disproportionately vulnerable to measles, and they are especially at a higher risk in low immunization settings. Household size of 4 turns out to be a threshold for the switch in vulnerability. The test results for household income reflect the effect of the difference in the assumptions for the uniform and the weighted decline in immunization rate.

## Discussion

In the past, studies have tried to estimate the impact of reduction in vaccination rate [10, 45], clusters of unvaccinated individuals [25], home isolation interventions [46] and speed of public health response [47] on measles outbreak and its prevention and control. Gaythorpe et al. [10] study the impact of disruptions due to COVID-19 on the increased risk of measles. However, these studies, when not based on mean-field models, have been performed on stylized networks, which do not consider detailed mixing patterns, especially in schools. In this study, we apply the disease transmission dynamics on a realistic and dynamic social contact network to analyze the threat of declining MMR vaccination rate on the measles outbreak. Novel aspects of our study are the highly resolved representation of school level contacts, diverse kinds of scenarios for reduction in vaccination rates, and the use of a high-performance-computing based simulation tool, which enables analysis of the variance and probability of large outbreaks.

**Fig. 8:**
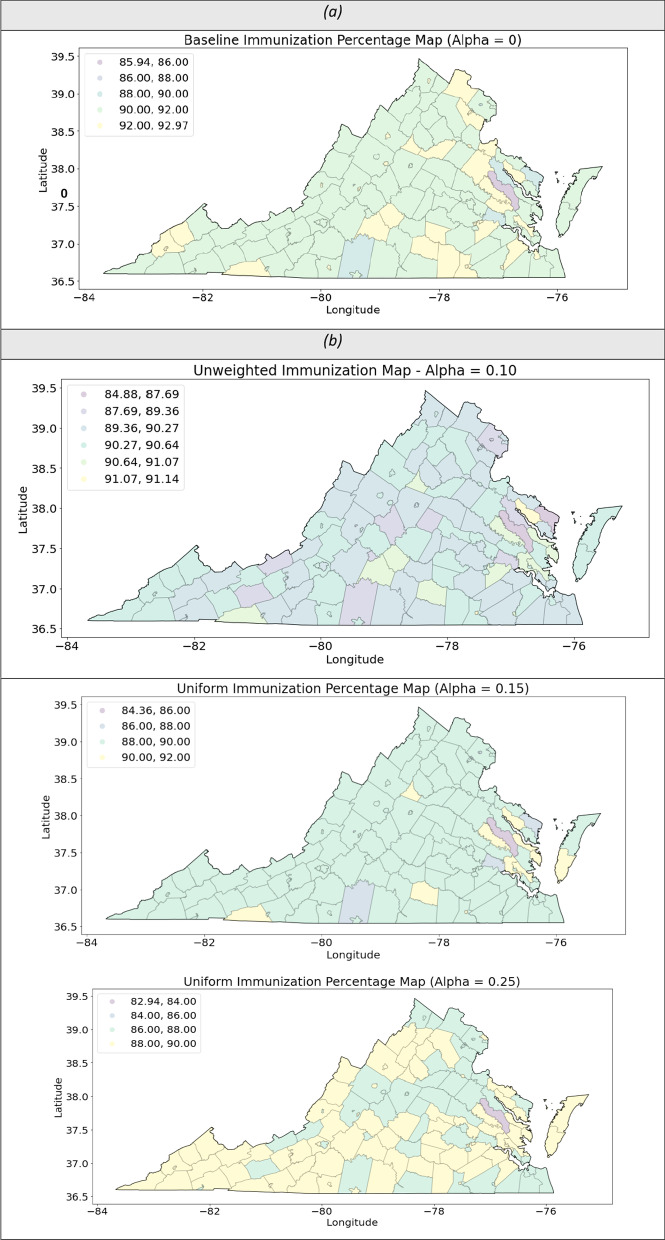

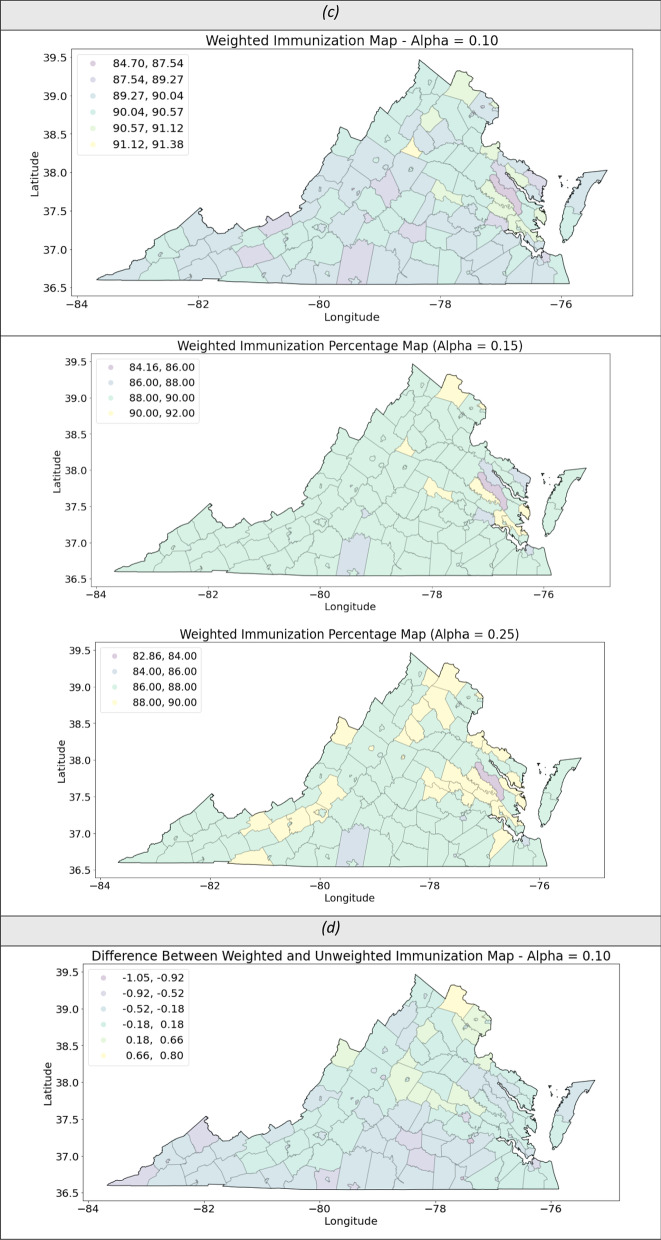

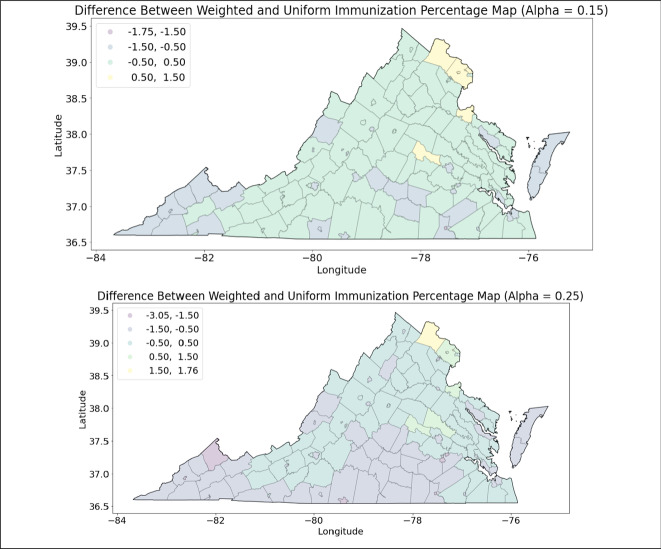
Decline in MMR rates. Spatial (county-wise) distribution of MMR immunization rates in the (a) base case ($\mathrm{\alpha }=0$); (b) post-COVID scenarios ($\mathrm{\alpha }=10, 15, 25$) when the decline in the immunization is random uniformly distributed over the population; (c) post-COVID scenarios ($\mathrm{\alpha }=10, 15, 25$) when the decline in the immunization is random uniformly distributed over the population when it is correlated with the household income (“weighted”); (d) the difference in the two types of distributions by county

In the post-lockdown era, the threat of measles resurgence is real as the schools and businesses reopen, while individuals are not caught up on their routine MMR immunization [3, 48]. The recent tragic measles outbreak in Afghanistan makes us wary of the risks of sub-par MMR immunization rates [49]. In 2017, Lo and Hotez showed that a 5% decrease in MMR vaccination resulted in a threefold increase in the number of cases [45]. Our results show that the decrease in vaccination rate $(\mathrm{\alpha })$ has a highly non-linear effect on the number of measles cases: a 5% decrease in vaccination rate results in a twofold increase in measles cases when the drop in the vaccination rate is concentrated in low-income households. When the decline in vaccination rate is 10%, we observe a more than tenfold increase in the weighted case but not so much in the uniform case. However in both scenarios this impact grows exponentially with additional decline in the vaccination rate. Note that reduced vaccination is evenly distributed in the uniform case but more spatially concentrated in low-income regions in the weighted case. At very low levels of RI, this difference has a small impact on the disease outcomes but at α = 10, this difference becomes significant. It is likely because spatial clusters of unvaccinated individuals cause a larger outbreak in the weighted scenario compared to the uniform scenario. At α >  = 15, the probability of outbreak becomes almost equally high in both scenarios, likely because the absolute number of unvaccinated is high enough in both cases to easily cause a large outbreak. Overall, a larger α translates into a larger risk under the weighted scenario compared to the uniform scenario. Please see Fig. 8a-d in Appendix corresponding to α = 10.

Additionally, the probability of having more than 1000 case counts also increases exponentially with $\mathrm{\alpha }$. In summary, as $\mathrm{\alpha }$ increases, the probability of a large outbreak with a single case importation becomes large, no matter where the reduced immunizations occur.

Based on the assumptions in the pre-COVID setting (base case), in our model, the resulting childhood MMR immunization rate for Virginia is 96.331% which is within the reported range for Virginia by the CDC [50]. The lockdowns and crises due to COVID-19 pandemic has resulted in the decline of routine immunization rates. The drop in children’s MMR vaccination rates is reported to be as high as 50% in some states neighboring Virginia, while the data for Virginia was unavailable [51] at the time of drafting this paper. We find that a decline in vaccination rate beyond 15% in Virginia will increase the potential for a measles outbreak by orders of magnitude (Fig. 3).

In the base-case scenario, that is pre-COVID vaccination rates, although on an average it does not seem like the effect of interventions makes a significant difference, we show that an additional day’s delay in initiation of home isolation increases the chance of an outbreak being bigger than NYC’s outbreak by about 5% (Fig. 5). Interventions that reduce the effective transmissibility beyond a threshold (0.7 in our case), will effectively decrease the risk of a large outbreak by as much as 10%. However, similar to the findings in [25], we find that in low immunization settings (more than 15% drop in the vaccination rate compared to the base case), interventions such as faster and higher compliance to home isolation will not be able to compensate for the risk of outbreak caused by the low vaccination coverage (Fig. 6).

The observation that increasing compliance to home isolation from 75 to 95% does not make a significant difference (only about 2%) in the probability of a large outbreak (Fig. 5) may be attributed to no change in behavior of the household members of the isolating individual, and to the transmission during the presymptomatic infectious period before isolation is initiated. Therefore, home quarantine for the contacts of the diagnosed individual, especially for the household members, is recommended for a higher effectiveness of the intervention.

Our results show that the potential size of an outbreak is similar whether the disease originates in a rural or an urban county when the vaccination rate is sufficiently high. This may be concerning to the public health officials since rural regions are typically not as well equipped with healthcare resources as the urban ones are if the spread is spatially concentrated. This is another reason for addressing disparities in access-to-healthcare in the rural regions.

Average outbreak size from a seed in a rural county is significantly smaller than the one caused by a seed in an urban county in the post-lockdown low-vaccination scenario. This may be due to the lower population density and mixing of the residents in the rural community as compared to the urban counterparts. However, the proportion of the rural and the urban communities getting infected is remarkably similar (Fig. 4); emphasizing that even if the absolute case counts are different, the proportional disease burden is similar. Therefore, proportional public health resources must be ensured which will be essential to the preparedness objective of World Health Organization’s strategic response plan for the upcoming decade [52]. In low vaccination rate settings, children under the age of 5 tend to be disproportionately infected. Since children are eligible for the second MMR dose only after the age of 4 years, this age group is particularly vulnerable.

Furthermore, to prepare, predict and prioritize the targets of interventions accurately, one must understand the causes of the decline in the immunization and identify the communities most vulnerable to the outbreak (top row of Fig. 4 shows difference in the rate of change of the order of magnitude of the outbreak size with the weighted and the uniform decrease in immunization). We see threshold behavior with the threshold values being different for the urban and the rural regions. Whether or not the African American population and the Native American population will be more vulnerable depends on the distribution of the decline of the vaccination rate (Fig. 7).

### Limitations

Most papers only study the measles transmission dynamics for the children (below 18 years of age); however, we chose to model unvaccinated adults too due to their contribution to the case counts in the NYC outbreak [2]. The following are some of the limitations of the work. We assume that the vaccine is perfect. Some studies have reported vaccinated individuals contracting measles [53–55], however most such cases are mild and have very few to no secondary infections [24]. The adults’ immunization rate estimate, due to lack of better available data, does not incorporate the migration of adults and hence may not be accurate. Nevertheless, migration of either vaccinated or unvaccinated adults in majority is unlikely. Additionally, per the resultant immunization rate in the synthetic population, children have a higher immunization rate (96%) compared to adults (90%) in our model which would cause transmission to occur more in adults. Our usage of the term “outbreak size” refers to the cumulative incidence within the one year of simulation, irrespective of whether the outbreak ended within the year or not since we aimed at measuring the risk over a limited period. Our model assumes that individuals in the post-lockdown era will resume activities at pre-covid levels which may not be true since work-related mobility and travel patterns may have been permanently altered.

There are many directions this work can be extended to. The model can be extended by incorporating other public health interventions like post-exposure prophylaxis and home quarantine through contact-tracing [56] and school closures [57]. It could also be extended to study the spread of measles in other states in the US or even at the national level. A look into the effect of delayed immunization campaigns over a longer time horizon as in [10, 58] would be insightful in quantifying and optimizing the economic burden. A positive birth rate, that dynamically adds to the susceptible pool, could be modeled to assess the implicit effect of spatial and demographic differences in birth rate on the likelihood of incidence of Measles.

## Conclusions

This research shows that as the immunization rate decreases, the probability of a large outbreak caused by a single case importation increases. In fact, the effect of the decline in immunization rate has a non-linear effect on the number of cases, and there exists a threshold beyond which the effect is exponential. In high-immunization settings, early interventions and home isolation strategies that reduce the transmission decrease the chances of a large outbreak. However, in low immunization settings, like the one in post-COVID-19 era when there is a drastic drop in the vaccination rate among children, isolation strategies (resulting in a reduction in transmissibility) are not enough to control the outbreak. A larger compliance to interventions or prompt action in terms of isolation are not sufficient to counter the effects of the additional drop in the vaccination rate. Identifying the cause of the decline in vaccination rates (e.g., low income) can help design targeted interventions which can dampen the disproportional impact on more vulnerable populations. Public health campaigns to raise awareness about the risks of measles and its prevention through vaccination can be a crucial tool in maintaining high rates of MMR vaccination. Engaging community leaders who can spread the message and arranging mobile vaccination clinics in low-income communities to provide easy access to care can also help in improving vaccination rates. This study also emphasizes the need for proportionally equitable public health resources in rural regions given that the per capita burden from reduced immunization is expected to be similar in rural and urban regions.

## Data Availability

The datasets are available from the corresponding author on request.

## Appendix

### HPC (high performance computing) requirements and usage

- Cluster used: Rivanna HPC Facility at University of Virginia
- Experiments run: 53
- Replicates per experiment: 300 (therefore: 15,900 simulations in total)
- Input
  - Static social network (synthetic population) data: 712 M
  - Dynamic contact network: 24G
- Output
  - Raw output (per simulation): 150 M—180 M
  - Output Summary (per simulation): 90 K—110 K
- Hardware
  - Launching static social network on a server: 375G (for a period of 48 h at a time, over 40 CPUs)
  - Resident set size (per simulation): 7G
  - Disk read/write size (per simulation): 2.5 M
  - CPU time used (per simulation, per 20 nodes): 1 h to 4.25 h

**Remark****: **Due to a relatively sparse number of cases in base care scenario outbreak leading to the high variability, the choice of at least 300 replicates is validated by the intuitive monotonic trend of average outbreak sizes upon varying different parameters.

### Weighted and uniform immunization maps

See Fig. 8.

### Calibration of the transmissibility parameter (${\varvec{\tau}}$)

Being the latest large outbreak with available data, we use the 2018–2019 measles outbreak data for New York city (NYC) to calibrate the transmissibility parameter $\tau$. We scale the monthly new cases to be proportional to the population size of Virginia (that is, $\#cases \left(VA\right)\approx \frac{\# cases (NYC) }{population size \left(NYC\right)}\bullet population size (VA)$). We perform a parameter sweep on the values of $\tau$ from 0.05 to 0.9 and obtain that for 300 replicates, $\tau =0.5$ assumption results in the adjusted monthly cases to fall with one standard error of the simulation outcome.

## Footnotes

1 http://www.here.com

2 http://www.nces.ed.gov

3 http://landscan.ornl.gov

4 http://www.openstreetmap.org

5 https://nces.ed.gov/ccd/

6 https://www.cdc.gov/vaccines/imz-managers/coverage/childvaxview/data-reports/mmr/trend/index.html
